# Observations on the Modus Operandi of Opium

**Published:** 1802-06

**Authors:** M. Ward

**Affiliations:** Manchester


### *Obſervations on the Modus Operandi of Opium.*

[ Continued from Vol. VII. p. 360. ]

WHEN it is conſidered what an amazing number of experiments have been made with Opium upon different animals, all tending to prove, that, however applied, it directly weakens, and ultimately deſtroys, all the different faculties and functions of the body; that it is remarkable above all other medicines for *inducing* ſleep, *which is prevented* by *ſtimulants*,* and that its characteriſtic quality is that of *allaying* every kind of *ſtimulus*,† it cannot but appear ſurprizing, that ſo many clear and poſitive proofs as are upon record, of its acting directly as a ſedative, ſhould have been ſet aſide, and that it ſhould have been pronounced a ſtimulant, in oppoſition to the moſt deciſive evidence, on account of its being often found primarily to increaſe the frequency and fulneſs, and ſometimes the ſtrength of the pulſe, and producing a few other effects,‡ which can only be conſidered as equivocal ſigns of a ſtimulant operation, and which will be found upon inveſtigation, *to proceed from the directly ſedative effects of the opium upon the different coats and veſſels of the ſtomach and ſmall inteſtines, independently of its action upon the nerves of theſe organs.*§ And

---

* Silence and repoſe, with a freedom from every ſenſation of *ſtimulus*, are no leſs effectual in producing ſleep. Sleep is impeded by every kind of *ſtimulus* of the ſenſes, or of the mind." Young on Opium, p. 24, 25.

† " It ſeemed to abate the preſent *ſtimulus* till the acrimony was corrected. The *ſtimulus is abated for a time*, by opium; but ſoon returns with more violence, as long as the acrimony continues. In this caſe the *ſtimulus* was ſo great that opium could not abate it. But when Nature is not able to bear the inceſſant *ſtimulus* and evacuation, it may be neceſſary to have recourſe to opium to procure an abatement of the ſymptoms, and ſome intervals of eaſe. Such ſpaſmodic contractions are increaſed by every effort of Nature to expel the ſtimulus. It may ſeem a kind of paradox, that the medicine which, *in the opinion of the world*, is thought to be chiefly uſeful as it *abates a ſtimulus*, ſhould yet be the beſt promoter of that ſtimulus which excites labour pains," &c. &c. Young's Treat. p. 33, 47, 48, 49, 57, 61.

‡ Such as, " In twenty minutes perceived a ſlight warmth, and ſoon after a degree of moiſture on my ſkin. In half an hour I found myſelf, *or at least imagined myſelf*, more alert and ſprightly than before. In twenty minutes my pulſe was fuller as well as quicker, the heat of my body was raiſed, and I perceived a manifeſt increaſe of ſpirits. In this ſtate I ſat down to dinner; and it was obſerved by one who knew nothing of what I had taken, that I looked as if very drunk," &c. &c. Inquiry, p. 35, 66, and 67.

§ It is not here ſuppoſed that the nerves of the ſtomach are exempt from the action of the opium, but that the effects are *chiefly* owing in this, *as well as in every other inſtance*, to its coming in contact, or nearly ſo, *with the arteries, and other veſſels, of the part to which it is applied.*

upon this idea the phænomena above enumerated, as well as feveral others, which have hitherto been confidered (and as long as Opium is fuppofed to act as a ftimulant will certainly remain) inexplicable, may be accounted for in a confiftent and fatisfactory manner; fuch as its allaying the cravings of hunger, producing naufea and vomiting; its fometimes *immediately* removing fuch diforders of the ftomach and bowels as arife from, or are accompanied with, exceffive irritability, as vomiting, diarrhœa, tenefmus, &c. &c.

*If opium acts in the way I have stated, i. e. solely and directly as a sedative, it must immediately diminish the sensibility and irritability of the arteries (those destined for secretion as well as nutrition) veins, absorbents, nerves and lacteals (the vessels and fibres, in short, of every description) of the stomach and upper part of the intestinal canal, and thereby their motions and powers of motion will be lessened or destroyed;* * *consequently, the muscular coat of the stomach and small intestines will act feebly, or not at all; and thus their peristaltic motion will be more or less impeded; hence vomiting, diarrhœa and tenesmus will be suppressed; the secretion of the gastric juice being also interrupted, and the process of digestion suspended or carried on imperfectly, the sensation of hunger will be allayed; the stomach being by the causes above-mentioned rendered incapable of performing its functions, its contents will advance rapidly towards such fermentations as their nature, whether animal or vegetable, renders them most liable to; hence nausea will take place, and continue, in a greater or less degree, till the stomach, stimulated by the acrid and offensive matter it may contain, acting mechanically, and probably also chemically, upon its tunics, will frequently recover its power* (where the dofe is not fo large as to *destroy* the fenfibility) *so far as to enable it to expel the whole or a part of its contents by vomiting.* †

As the arteries of the ftomach are both numerous and confiderable, and have frequent communications with the arteries of

* Precifely in the fame manner as when it is applied to any other part of the body.

† " In a fmall dog which Dr. K. Boerhaave opened after having given him three grains of opium, he obferved fcarce any periftaltic motion in the guts; *the stomach was much distended, the pylorus was shut, and the bread and milk, which the dog had taken with the opium about ten hours before, was indigested. There was nothing like chyle in the duodenum, nor any lacteal vessels to be seen in the mesentery. The bladder of urine and great guts were* much filled, nor had the animal evacuated either urine or fæces from the time he fwallowed the opium." Whytt's Effay, p. 345.

of all the neighbouring vifcera*, they muft require a pretty large quantity of blood to fill and circulate through them; and if the regular demand thus made upon the heart be fuddenly leffened, (*as it certainly is in a greater or lefs degree when opium is taken internally*, by its diminifhing their fenfibility, irritability, and mobility, in confequence of which the regular tranfmiffion of blood, through all thofe branches which are near enough to be influenced by the opium, is interrupted) it will have the effect, for a fhort time, of increafing the frequency, fulnefs, and ftrength of the pulfations of the heart and arteries, and thefe effects will be more or lefs confiderable, and of longer or fhorter duration, according to circumftances; but if my ideas of the modus operandi of Opium be well founded, they will *always* occur (when they do take place) *in a very fhort time after it has been taken*; and this appears both from the experiments and obfervations of different writers to be actually the cafe †.

But to be more particular, let us fuppofe an adult fubject, unaccuftomed to the ufe of Opium, to have taken two grains, or two and a half; its *primary effect* I prefume will be, to diminifh the frequency and force of the pulfations of the fmaller feries of arteries fituated in and near to the villous coat of the ftomach, and by degrees (fooner or later in proportion to their vicinity, and to their ability to refift the power of the medicine) it will retard, and at length put a ftop to the circulation in the larger arterial branches, not only in the villous but in the mufcular and other coats of the ftomach, (it will at the fame time leffen or intercept the motion of the nervous power in the nervous coat of the ftomach, and may alfo have fome influence upon the nerves of the other vifcera), and the heart and large arteries, being unable to overcome the refiftance, a degree of plethora will take place, pro tempore, which will increafe the frequency, ftrength, and fulnefs of the heart's contractions; an additional quantity of blood being now fent

* "From this detail we learn, that the arteria coronaria ventriculi, pylorica, inteftinalis, both gaftricæ, gaftro epiploicæ, and confequently the hepatica, fplenica, and mefenterica, communicate all together." Monro's Syftem of Anat. Vol. 3, p. 130.

† "In Dr. Crumpe's fixth and eighth experiments the pulfe was found to be increafed *in five minutes* from 70 to 74, and in his feventeenth from 70 to 78 in the fame time; in the nineteenth 15 minutes were required to raife it from 72 to 74; but in this experiment he had taken the gum *freed from the refin*: and in the feventh experiment, 25 minutes had elapfed before the pulfe became more frequent, which will not appear furprizing when we confider that one grain had been taken by a robuft healthy young man, the natural ftandard of whofe pulfe was no more than 44 pulfations in a minute.

ſent to the brain, as well as to the other parts of the body, its energy will be increaſed, and he will feel himſelf more or leſs exhilarated; probably alſo, the heat and perſpiration may be ſomewhat augmented. But it will be obvious that this ſtate of things cannot continue a long time, ſeldom longer I imagine than 30 or 40 minutes) becauſe, in general, by this time, the circulation will have ceaſed *in the veins* of the different coats of the ſtomach *, and whenever that happens, a ſmaller quantity of blood will be ſent to the right auricle of the heart, which will immediately take off the plethora, and reduce the frequency, ſtrength, and fulneſs of the pulſe, from which cauſe a degree of languor will be perceived; by this time alſo a part of the Opium will in all probability have been conveyed to the heart, by the lacteals or abſorbents of the ſtomach and inteſtines; and this concurrence of circumſtances will have a powerful effect in diminiſhing the frequency, ſtrength, and fulneſs of the heart's contractions, *so as to reduce them conſiderably below the natural ſtandard*; and the general excitement of the ſyſtem being in this manner ſuddenly diminiſhed, the languor will now be more conſiderable in proportion to the want of vigour in the circulation, and will be accompanied by drowſineſs, vertigo, nauſea, pain in the head, tremors in the hands, &c.; ſome, or all, of which ſymptoms will continue to be more or leſs troubleſome, till the veſſels of the ſtomach and inteſtines have recovered their ſenſibility, irritability, and mobility; and in proportion to the rapidity of this proceſs, will the ſtomach and inteſtines be reſtored to the exerciſe of their natural functions: the pulſe will ſpeedily reaſſume its uſual frequency and ſtrength, when the veſſels of the ſtomach again become pervious, ſo as to allow a free paſſage to the blood.

But let us, in the next place, ſuppoſe a perſon in ſimilar circumſtances, to have taken *a large doſe of opium*, e. g. four grains or upwards; this quantity will operate ſo inſtantaneouſly and powerfully as a ſedative upon the ſenſibility, irritability, and mobility of the veins, as well as the arteries and other veſſels belonging to the ſtomach and inteſtines, and alſo upon the lacteals and ducts †, which open into the primæ viæ; and its influence will be ſo much more extenſive than in the former caſe, as to occaſion a conſiderable quantity of blood which was returning to the vena cava inferior from the ſtomach, the inteſtines, the ſpleen, the pancreas, the meſentery, *and*

* The coats of the veins being leſs irritable than thoſe of the arteries, and the circulation in them not depending principally, as in the arteries, on muſcular action, I imagine that *a moderate doſe of opium* will not ſo ſpeedily t a ſtop to the circulation in the former as in the latter.

† The ductus pancreaticus and communis choledochus.

*and more especially from the liver* by the hepatic veins, to be intercepted and detained in the veins belonging to the different viscera; and one great source by which the heart is usually supplied with blood being in this manner cut off, it will send out a smaller quantity at each contraction, and not being able, any more than the aorta and pulmonary arteries, immediately to adapt itself to the diminished quantity of blood, the latter will not be propelled with the same force as before, which will speedily retard, if not interrupt, the circulation in the minute vessels of the extremities; as we have seen exemplified in the experiments of Dr. Alston.

If the heart sends out a smaller quantity of blood than before, less will be sent to the lungs in a given time, and the quantity sent will not be transmitted with the same facility as before, on account of the inactivity of the pulmonary arteries, which will soon have the effect of retarding the circulation in the pulmonary veins, and thus, one of the most important purposes of respiration, namely, the oxygenation of the blood, will be imperfectly executed, which will add to the debility already prevailing in the system. The passage of the blood from the right ventricle to the left auricle being in this manner obstructed, a degree of uneasiness will be excited in the chest, which will be a little relieved by every inspiration and expiration, (the alternate dilatation and compression of the lungs in respiration making up, in some degree, for the want of power in the heart and pulmonary arteries) he will therefore make deeper inspirations, and endeavour to put the intercostal and other muscles employed in respiration into action more frequently, with a view to remove the oppression; the relief obtained, however, will be only temporary; for the torpor in the part first exposed to action of the opium having been, by this time, communicated in a greater or less degree to the diaphragm, the intercostal and abdominal muscles, the respiration will gradually become slow, difficult, and laborious; and as the tendency to congestion increases, possibly some of the veins may give way so as to allow the blood to escape into the neighbouring cells, still farther impeding respiration, and contributing (with another cause to be mentioned by and by) to render it stertorous as well as laborious.

The circulation in the veins having lost much of that assistance which it commonly receives from the arteries, when their pulsations are strong and vigorous, and the torpor continuing to increase throughout the system, the vena cava will convey less and less blood to the heart, and the circulation will become more and more languid; and now, from the want of energy in the vessels of the brain, arising from the diminished power in the heart and and arteries, the blood will loiter and ſtagnate in the veins and ſinuses of the head, and occaſion coma, ſtertorous breathing, ſubſultus tendinum, &c. Should any of the veins or ſinuses give way (which may very probably happen, partly from a loſs of tone in the veſſels, and partly from their being more diſtended with blood than uſual) convulſions, when they occur at this late period, may ariſe from this cauſe; but they in general occur in a much ſhorter time after the Opium has been exhibited than we are at preſent ſuppoſing; and in ſuch caſes, *I attribute them to the Opium deſtroying the mobility of the vaſcular system, in a greater degree, and more ſpeedily, than the mobility of the nervous power.**

As an illuſtration, and at the ſame time as a proof of the truth of what has been ſuggeſted, it may be worth while to compare the ſtate of the appetite and the circulation in a few hours, or in any given time, after a doſe of Opium that has been taken internally, with the ſtate of theſe functions when an equivalent portion has been applied externally.

If

* The following obſervations appear to me to throw conſiderable light upon this part of the ſubject. "That opium cauſes convulſions is owing, in my opinion, to its deſtroying at different times, and in an irregular way, the irritability of the muſcular fibres. It is beſides certain that men and women of a delicate and weak frame are always the moſt ſubject to convulſions; and it is not poſſible to ſuppoſe in theſe people a ſuperabundance of animal ſpirits. We know that all the muſcles, even in a relaxed ſtate, preſerve notwithſtanding a certain tenſion of their fibres, which, when they are cut, never fail to contract themſelves, and to enlarge the wound. When a muſcle becomes paralytic, it lengthens, and its antagoniſt then contracts the more; which ſhows that the repoſe of the muſcles depends on the equilibrium of ſtrength betwixt the different muſcles, and betwixt their different fibres. The powers thus balanced, deſtroy and renew themſelves at every inſtant, without producing any motion or ſenſible change. This natural tenſion of the muſcular fibres certainly depends on an equal and exact diſtribution of the fluids in the whole ſubſtance of the muſcles. This truth is demonſtrated in a Diſſertation which I publiſhed in the third vol. of the Acts of Sienna, which was in part reprinted ſome time after at Lucca, with ſeveral conſiderable additions, and which was afterwards inſerted in the firſt vol. of my Animal Phyſics. But if theſe muſcles do not receive the ſame proportion of fluids, or if theſe fluids reach them, or are diſtributed amongſt them, with an unequal quickneſs and energy, the equilibrium of the mutual effort of the muſcles is immediately deſtroyed; the ſtrongeſt of them contract; and hence ariſe the convulſions and violent agitations of the whole frame. This is the reaſon why thoſe who die of an hæmorrhage, as well as thoſe who periſh by poiſon, are ſeized with convulſions; for it certainly is not probable that the loſs of blood and of ſtrength ſhould bear an equal proportion in every part, in every muſcle, and in every fibre, whilſt the circulation itſelf is unequal, and the muſcular irritability is deſtroyed gradually, and in a very irregular way, according to time and circumſtance." Fontana's Treatiſe on Poiſons, Vol. I. p. 81. 82.

If Opium produces any one effect more regularly than another in the former mode of exhibiting it, namely, by the mouth, it is that of *impairing* the appetite and digeftion; and it often, as we have feen, *increases* the frequency of the pulfe. But in the latter mode of applying it, the effects are exactly reverfed, that is, the appetite and digeftion are *never* impaired, *but often improved and strengthened, and the frequency of the pulse is diminished.*

The caufes are obvious and eafily explained, if we only keep in mind that in the mode of exhibiting it firft fuppofed, it comes into contact with the internal furface of the ftomach and inteftines, but muft pafs through a circuitous courfe before it arrives at the heart and arteries; and that in the other mode of introducing it, namely, by the lymphatics, it has immediate accefs, or nearly fo, to the heart and arteries, but has no immediate communication with the alimentary canal.*

Thus

---

* The manner in which I fuppofe it to operate when it improves and ftrengthens the appetite and digeftion, is by diminifhing the fenfibility, irritability, and mobility of the heart and arteries, and thereby difpofing them to receive and retain a larger quantity of chyle, than the irritable and contracted ftate they were in before would admit of.

By viewing the fubject in this light, I am led to believe that the opiate frictions may be of fervice in the treatment of phthifis pulmonalis, as foon as the hectic fever is fully formed, when the taking of nutriment has in general the effect of creating a good deal of difturbance in the fanguineous fyftem of veffels, by which the debility is increafed in the fyftem at large, and a greater determination of blood to the lungs than ufual takes place, increafing inflammation in the thoracic vifcera where it is prefent, or furnifhing a predifpofition to it where it does not already exift.

The proper times for their application would be, I fhould conceive, immediately after every meal, *or at least after the principal meals*; but a good deal would depend upon the degree of excitement which the food might occafion.

Should the difturbance be prevented, *and the frequency of the pulse reduced by these means*, the patient might then, with great probability of advantage, ufe fuch moderate exercife as might appear to be beft adapted to the degree of bodily ftrength he poffeffes; as riding in a carriage, on horfeback, or walking.

The *internal* ufe of opium I fhould fuppofe to be highly improper *in every stage of the disease*, on account of its tendency to impair and deftroy the functions of the ftomach and inteftines, (by diminifhing their action and fecretions) and thus often increafing the mobility of the vafcular fyftem, as well as the fweating, in each of thefe ways increafing the debility; and this idea fhould be conftantly kept in view with regard both to diet and medicine.

It may not be altogether foreign to the fubject to mention an opinion I have long entertained, that opiate frictions may probably be of ufe in fome circumftances of the treatment of moft if not all thofe difeafes which form the third Order of the fecond Clafs of Cullen's Nofology, and have feveral times, particularly in my firft paper, publifhed nearly three years ago, expreffed

Thus it appears, that upon the principles laid down above, the various and insuperable difficulties which occur in every other

---

preffed a firm conviction of their probable utility in hydrophobia and tetanus. They have been tried with advantage in the latter, and it now appears that a good deal of benefit has been derived from their ufe in pertuffis and cholera, (fee the laft Number of the Medical Journal, p. 305,) all which difeafes belong to the clafs and order abovementioned; but there is another moft formidable and in general fatal difeafe, belonging to the fame order and clafs, in which I think they promife, *with the assistance of opium internally*, to be extremely beneficial. The difeafe to which I allude is diabetes.

It is with great diffidence that I venture to fubmit the following opinions to the ferious and candid confideration of the readers of the Medical and Phyfical Journal.

I fhall not hazard an opinion as to what may in general be the remote caufe of this difeafe; I fufpect, however, that it fometimes arifes from expofure to cold, accompanied by violent or unufual exercife.

The proximate caufe I fuppofe to confift in an increafed action of all the veffels and coats of the ftomach, *occasioning an increased secretion of the gastric juice.* I conceive alfo, that the unufual quantity of gaftric liquor fecreted, ftimulates the internal furface of the lacteals and abforbents of the ftomach and inteftines, as well as that of the arteries, veins, and emulgent veffels, and thereby increafes their mobility, and produces the immoderate difcharge of urine, and alfo the great thirft and voracious appetite; and this I imagine to be the ftate of the natural and vital functions in diabetes infipidus.

In diabetes mellitus, I imagine there is not only an *increased secretion* of gaftric liquor, but that there is alfo an alteration in its properties.

If the above reprefentation be founded in truth, (which I believe it to be) it feems to me to fuggeft a different mode of treatment to any that has hitherto been recommended. The indications will be fimple and clear, viz. to evacuate the gaftric juice already fecreted in the ftomach; fecondly, to diminifh the action of the veffels and coats of the ftomach, and thereby the fecretion of the gaftric liquor; and, thirdly, to obviate or remove the fymptoms.

The difficulty will confift in devifing the moft proper means by which the indications may be beft fulfilled. I fhall deliver my fentiments in a few words; and I expect that the opinion I have formed of the Modus Operandi of Opium, will be of fome practical utility on the prefent occafion.

With a view to the firft indication, the treatment fhould commence with moderate vomiting, and for this purpofe tartarized antimony feems preferable to ipecacuanha, becaufe it produces a greater degree of naufea as well as diaphorefis; and the moft proper time for adminiftering it feems to be, when the fenfation of hunger or thirft is moft keen, as there would then be a fair profpect of evacuating a portion of the gaftric liquor.

In order to diminifh the fecretion of gaftric juice, which forms the fecond and *principal* indication, the fenfibility, irritability, and mobility of the veffels and coats of the ftomach muft be diminifhed; and I know of no medicine fo powerful as opium, *internally administered,* in producing thefe effects; but then it fhould be given in fuch dofes as will *not* fo *speedily* diminifh their mobility, as to *increase* the frequency of the pulfe; as from three-quarters of a grain to a grain and a quarter, or from fifteen to twenty-five drops of tincture of opium, three, four, or five times in the twenty-four hours; the beft other theory of the modus operandi of opium, *and particularly in the stimulant theory*, entirely vanifh, and all the phenomena may be explained in a fimple, confiftent, and fatisfactory manner; without the neceffity alfo of reforting to the improbable fuppofition of the exiftence and operation of a vis medicatrix naturæ.

But in endeavouring to explain the modus operandi, I would not be underftood to affert that each fymptom occurs precifely in the order and degree I have ftated. We know, for inftance, that naufea and conftipation are not *always* the confequence of

---

beft time to adminifter the medicine appears to be when hunger or thirft is moft prevalent, and at bed-time.

Its fedative effects however fhould be guarded againft, and coftivenefs obviated by oleum ricini internally, or by laxative clyfters. Its effects upon the circulation fhould be afcertained by attentively examining the pulfe; as this will be the beft criterion by which to regulate the dofe, and the frequency of its repetition. If the frequency of the pulfe is not materially increafed primarily, *nor much diminished ultimately*, the dofe might be either fomewhat augmented, or repeated rather oftener, when the hunger or thirft is preffing, or the difcharge of urine not leffened.

Would its efficacy be increafed by joining with it fmall dofes of tartarized antimony; or of ipecac. as in the pulv. ipec. comp. or would nitre and tartarized antimony be preferable in fome cafes?

The fymptoms which are generally moft troublefome, are hunger, thirft, and the frequent and copious difcharge of urine.

The two former have already been in part attended to, in fpeaking of the moft proper means to be ufed for anfwering the fecond indication; *and as opium diminishes all the secretions except that of sweat*, it feems admirably adapted to every circumftance and fymptom of the difeafe.

Whether the internal or external application acts moft powerfully in diminifhing the fecretion of urine, remains to be tried; but where the difeafe is accompanied by fpafmodic affections, and thefe are not relieved by the internal ufe of opium, or where hectic fever is prefent, I fhould be ftrongly inclined to recommend the *external* application, as in phthifis.

In conformity with the above idea of the proximate caufe, an attention to diet will form a very effential part of the treatment.

It fhould chiefly confift of fuch kinds of animal food as are moft difficult of digeftion; young meats of every defcription fhould therefore be avoided, and boiled beef, mutton, pork, tripe, &c. be preferred.

The drink ought principally, I think, to confift of milk both at meals and other times; becaufe it is not always eafily digefted and is frequently coagulated by the gaftric juice, which may blunt and diminifh its ftimulating qualities; but occafionally, a moderate quantity of brandy and water, or ftrong green tea, might perhaps be allowed.

The above plan of treatment, judicioufly varied according to circumftances and emergencies, feems to me to promife much; fhould it fail, we muft ftill I think rely principally, if we expect to fucceed in curing the difeafe, upon that clafs of medicines *ranked as narcotic sedatives by Dr. Cullen*, with the exception of tobacco, wine, and alcohol; and there can be no impropriety or danger in making a *cautious* trial of fuch medicines of this clafs, as may appear, from their known properties, to be beft adapted to the indications.

 taking taking opium internally; and that when they do occur, they sometimes take place at an earlier, sometimes at a later period; and the same remark will apply to some of the other symptoms, such as convulsions, coma, laborious respiration, &c.

*Manchester, April* 8, 1802. M. WARD.

[ To be continued. ]

